# Regional investigation of UT-B urea transporters in the rat brain

**DOI:** 10.1016/j.bbrep.2023.101563

**Published:** 2023-10-25

**Authors:** Farhana Pinki, Derek A. Costello, Gavin Stewart

**Affiliations:** a UCD School of Biology and Environmental Science, University College Dublin, Dublin 4, Ireland; b UCD School of Biomolecular and Biomedical Science, University College Dublin, Dublin 4, Ireland; c UCD Conway Institute, University College Dublin, Dublin 4, Ireland

**Keywords:** Urea, UT-B transporter, Brain, Region, Protein

## Abstract

Recent studies have reported increased levels of urea in the aging brain and various neurological disorders. Additionally, these diseased tissues also have increased expression of the UT-B transporter that regulates urea transport in the brain. However, little is known regarding the actual UT-B protein distribution across the brain in either normal or diseased states. This current study investigated UT-B protein abundance across three regions of the rat brain – anterior, posterior and cerebellum. Endpoint RT-PCR experiments showed that there were no regional differences in UT-B RNA expression (NS, N = 3, ANOVA), whilst Western blotting confirmed no difference in the abundance of a 35 kDa UT-B protein (NS, N = 3–4, ANOVA). In contrast, there was a significant variation in a non-UT-B 100 kDa protein (P < 0.001, N = 3–4, ANOVA), which was also detected by anti-UT-B antibodies. Using the C6 rat astrocyte cell line, Western blot analysis showed that 48-h incubation in either 5 mM or 10 mM significantly increased a 30–45 kDa UT-B protein signal (P < 0.05, N = 3, ANOVA). Furthermore, investigation of compartmentalized C6 protein samples showed the 30–45 kDa signal in the membrane fraction, whilst the 100 kDa non-UT-B signal was predominantly in the cytosolic fraction. Finally, immunolocalization studies gave surprisingly weak detection of rat UT-B, except for strong staining of red blood cells in the cerebellum. In conclusion, this study confirmed that RNA expression and protein abundance of UT-B were equal across all regions of the rat brain, suggesting that urea levels were also similar. However, it also highlighted some of the technical challenges of studying urea transporters at the protein level.

## Highlights

• Confirmed equal UT-B RNA expression across rat brain regions.
• Determined equal UT-B protein abundance across rat brain regions.
• Identified important technical challenges specifically detecting UT-B proteins in the brain.

## 1 Introduction

Classically, urea was viewed as a toxic nitrogenous breakdown product of protein catabolism generated in the liver by the ornithine-urea cycle, which was then simply excreted in urine and feces. Understanding of the physiological significance of urea was revolutionized in the 1990s with the discovery of urea transporters [1]. Facilitative urea transporters in mammals are derived from Slc14a2 and Slc14a1 genes, encoding UT-A and UT-B transporters respectively, which are both located on chromosome 18 [2]. UT-B was first identified as the primary brain urea transporter when it was cloned from rat brain tissue [3]. Since then, UT-B protein expression has been reported in various other mammalian brains – including mice [4] and humans [5]. UT-B is believed to be essential for maintaining urea concentration in the central nervous system and may be important in balancing sodium and water in cerebrospinal fluid [6]. Our previous studies have also suggested a potential role for UT-B in regulation of inflammatory responses in the brain [7]. Importantly, knockout of UT-B transporters in mice produced significant consequences in the brain, including a substantial increase in urea concentration within the hippocampus and a display of depression-like behavior [8].

Many studies have reported that a complete ornithine-urea cycle is only present in the liver and, although some constituent enzymes may be expressed in other tissues including brain, there is no functional urea cycle there. Instead, the brain under normal conditions utilizes glutamine synthesis for the removal of waste ammonia [[9], [10], [11], [12]]. The production of urea is a critical aspect of protein catabolism, but increased levels of urea and its nitrogenous precursor ammonia are toxic to the brain, as seen in uremic encephalopathy [13]. Resulting from urea build-up in the blood and brain, this is common in patients with either kidney failure or urea cycle disorders, resulting in hyperammonemia because key enzymes lose function due to genetic variation [14].

Importantly, urea levels have been examined in diseased brain tissues in various species. For example, several regions in the human Alzheimer's disease (AD) brain showed alterations in metabolites involved in several biological pathways, including the urea cycle [15]. Indeed, urea was significantly elevated throughout the AD brain [15]. Increased levels of urea have also recently been reported in the aging human brain in the case of vascular dementia [16] and in Parkinson's Disease [17]. Furthermore, Handley et al. [14] demonstrated using a prodromal sheep model of Huntington's Disease that increased levels of urea increased UT-B RNA expression. Notably, postmortem Huntington disease human brain also showed increased urea levels, suggesting elevated protein breakdown as an energy source due to the metabolic dysfunction accompanying Huntington disease [14]. Crucially, altered brain UT-B transporter expression has also been identified in AD, Parkinson's disease and amyotrophic lateral sclerosis [18], along with a whole range of other brain-related medical conditions – including non-suicidal self-injury in adolescents [19] and chronic alcoholism [20]. However, what causes the urea increase, or whether this contributes to the pathology or not, is currently not understood. Nevertheless, Handley et al. [14] suggest that a potential therapeutic target in Huntington's disease could be reducing brain urea and/or ammonia levels, and this requires thorough investigations of the mechanism of urea cycle disruption and cause of elevated urea. Unfortunately, regional brain UT-B protein abundance or localization remains largely unreported.

Using rat tissue to study brain UT-B function, original studies detailed that UT-B RNA expression was prevalent throughout the rat brain [21]. Furthermore, UT-B1 proteins have been detected in the rat brain, mainly in astrocytes and ependymal cells [22] and their expression is shown to increase with aging [23]. Nevertheless, relatively little is understood regarding the specific regional variation of UT-B1 proteins in the rat brain. The aim of this current study was therefore to investigate the regional expression, abundance, and localization of rat brain UT-B urea transporters.

## 2 Methods

### 2.1 Animals

Adult male Wistar rats (5–8 weeks old) were euthanized with CO_2_ to provide tissue for undergraduate teaching activities. Postmortem brain tissue was subsequently isolated for research purposes in accordance with a tissue-sharing initiative, and following approval of the UCD Animal Ethics Committee.

### 2.2 RT-PCR

Total RNA was extracted using a standard isolation protocol with RNA-STAT 60 (AMS Biotechnology, UK), BCP, isopropanol, and ethanol. Extracted RNA samples underwent treatment with Ambion TURBO DNA-free kit (Thermo Fisher Scientific, USA) with Turbo DNase enzyme for 25 min at 37 °C before quantification with NanoDrop 1000 Spectrophotometer (Thermo Fisher Scientific, USA). cDNA preparation was performed using SensiFast cDNA synthesis kit (Bioline, UK) and employed prepared rat brain RNA samples. PCR amplification of the cDNA samples with Go-Taq polymerase and primers for PGK1 or UT-B1 was performed using a Biometra T3000 thermocycler (Biometra, Germany). Primers sequences were as follows: UT-B forward 5′-CCCTCTTGCTTAGCCAAGACAG-3’; UT-B reverse 5′- GAGGAGAGCAGGATAG CACATAG-3’; PGK1 forward 5′-GGTGGACTTCAACGTTCCTATG-3’; PGK1 reverse 5′-CTA AACATTGCTGAGAGCATCC-3’. Cycling parameters for PCR reactions involved initial denaturation at 94 °C for 4 min, followed by 35 cycles of denaturation at 94 °C for 30 s, annealing at 60 °C for 30 s, and extension at 72 °C for 30 s with a final extension at 72 °C for 10 min. Direct sequencing (Eurofins Genomics, Germany) was used to confirm identification of PCR products.

### 2.3 Antibodies

UT-B transporter proteins were detected using the previously characterized UT-Bc19 antibodies [24]. Commercial antibodies raised against GAPDH (Y3322GAPDH, AMS Biotechnology) and NaKATP (SC-48345, Santa Cruz Biotechnology) were also used. These primary antibodies were used in connection with horseradish peroxidase conjugated anti-rabbit (65–6120, Invitrogen) or anti-mouse (61–6520, Invitrogen) secondary antibodies as appropriate.

### 2.4 Cell lines

Rat C6 astrocyte cells were cultured in DMEM F-12 media (Lonza, USA) mixed with 20% heat-inactivated FBS (fetal bovine serum) and 1% penicillin-streptomycin. For urea treatment experiments, C6 cells were grown in 0, 5 or 10 mM urea for a period of 48 h prior to RNA and protein preparation. Human epithelial bladder transitional papilloma RT4 cells (ATCC, Manassas, VA) were cultured in McCoy's 5A modified medium supplemented with 10% heat inactivated foetal bovine serum. In both cases, the cell lines were grown in incubators set at 37 °C with 5% CO_2_.

### 2.5 Western blotting

Dissected brain tissue samples from male Wistar rats were initially homogenized with a Polytron PT1200 E homogenizer (Kinematica, Switzerland) and buffer (300 mM Mannitol, 12 mM HEPES, pH 7.6). Centrifugation of these homogenized samples was performed at 1000 g for 5 min at 4 °C, then the resulting supernatant was centrifuged at 16,300 g for 30 min at 4 °C, and the final pellet was re-suspended in fresh homogenization buffer, yielding membrane-enriched protein samples. For compartmentalization studies, homogenized RT4 and C6 protein samples underwent differential centrifugation in a series of buffers to separate them into cytosolic, nuclear, membrane and cytoskeletal fractions (CNMS Compartmentalization Kit, AMS Biotechnology). All protein samples were mixed at a 1:3 ratio with 4X Laemmli buffer [1% SDS, 10% glycerol, 31.5 mM Tris-HCl (pH 6.8), and bromophenol blue] and heated at 70 °C for 10 min before being loaded onto gels (∼10 μg per lane). SDS-PAGE was performed using Criterion TGX precast 8–16% protein gels (Bio-Rad, USA) and a Criterion cell tank (Bio-Rad, USA). The separated proteins were then transferred to a nitrocellulose membrane and left in overnight incubation at room temperature with primary antibody, in 1:1000 hUT-Bc19, 1:2000 GAPDH or 1:2000 NaKATP. Membranes were then washed and probed with 1:5000 horseradish peroxidase-conjugated anti-rabbit antibody or anti-mouse antibody for 1 h at room temperature. Detection of proteins were performed using Western Lightning Plus ECL reagents (PerkinElmer, USA) and a LAS-4000 Imager (Fujifilm, Japan). Peptide inhibition experiments were performed with hUT-Bc19 preincubated with specific or non-specific immunizing peptide for 24 h using a rotating mixer.

### 2.6 Immunolocalization

Rat brain tissue sections (5 μm in thickness) were prepared in the laboratory using a Rotary Microtome CUT 4060 (Microtec, Germany). Sections then underwent Neoclear treatment and a series of rehydration reactions with ethanol concentrations (100-70%). Endogenous peroxidase activity in sections was blocked with 0.3% hydrogen peroxide in methanol for 30 min and quenching of the sections was performed with 50 mM ammonium chloride in PBS for 30 min. Antigen retrieval was performed by boiling sections for 1 min in TEG (Tris-EGTA) buffer solution (1.21 g Tris HCl, 0.19 g EGTA, 1000 ml dH_2_O) before overnight incubation at 4 °C with either 1:100 or 1:250 dilution of hUT-Bc19 in 0.1% BSA and 0.3% Triton X-100 in PBS. Immunolabeling was visualized with a 1:1000 dilution of horseradish peroxidase-conjugated anti-rabbit secondary antibody in 0.1% BSA and 0.3% Triton X-100 in PBS followed by an incubation with diaminobenzidine and counterstaining with hematoxylin. Stained sections then underwent a series of dehydration reactions with ethanol concentrations (70–100%) and subsequent Neoclear treatment. Coverslips were mounted on sections using Eukitt mounting medium and stored at room temperature before obtaining detailed images using Leica DMLB microscope (Leica Microsystems, Germany).

## 3 Results

**Fig. 1:**
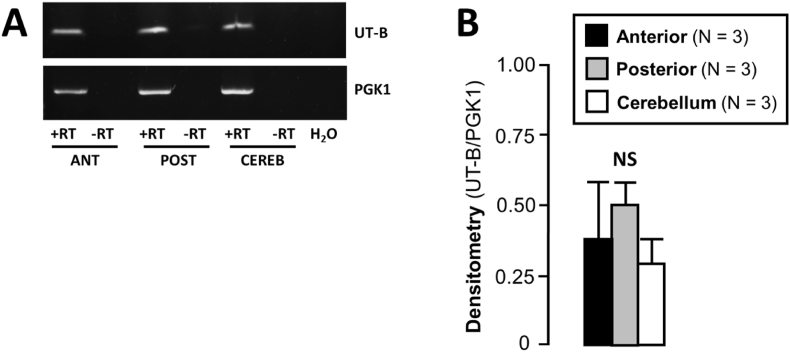
**RNA expression of PGK1 and UT-B in cDNA from rat anterior, posterior and cerebellum brain regions. (A)** RT-PCR of rat brain cDNA samples (∼1 μg per reaction) using UT-B and PGK1 control primers. Strong detection of the expected 1189 bp PGK1 control signals was observed in all samples. The 439 bp UT-B signal was also detected in all anterior, posterior and cerebellum brain samples. **Key**: +RT = + Reverse Transcriptase reaction; -RT = - Reverse Transcriptase reaction; ANT = Rat Anterior Brain; POST = Rat Posterior Brain; CEREB = Rat Cerebellum. **(B)** Bar graphs summarizing the results of densitometric analysis of UT-B and PGK1 PCR signals.

Initial endpoint RT-PCR experiments were performed to investigate UT-B RNA expression in three regions of the rat brain – anterior, posterior, and cerebellum. UT-B RNA expression was relatively consistent across all three regions (Fig. 1A), as was that of a control gene, PGK1. Densitometric analysis using UT-B expression normalized to PGK1 confirmed that there were no significant differences in UT-B expression across the regions (NS, N = 3, Repeated measures ANOVA) (Fig. 1B).

**Fig. 2:**
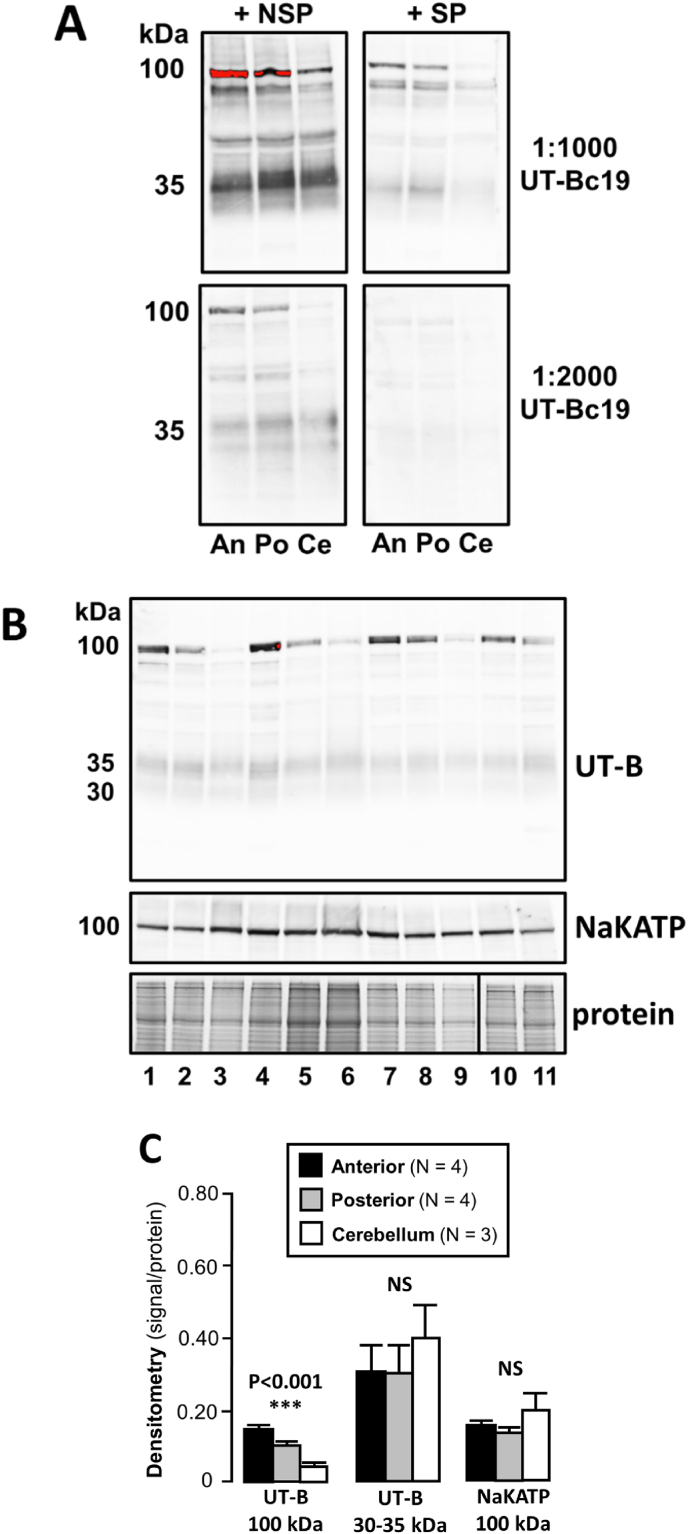
**UT-B protein expression rat brain regions. (A)** Peptide inhibition experiments using 2 μg/ml specific or non-specific peptides, with 1:1000 or 1:2000 UT-Bc19. These experiments showed that both the expected 30–35 kDa UT-B signal and the other 100 kDa signals were all visibly reduced by the specific immunizing peptide. **Key**: An = Anterior; Po = Posterior; Ce = Cerebellum; NSP = Non-specific peptide; SP = Specific immunizing peptide. **(B)** Western blots of rat anterior, posterior, and cerebellum brain samples (∼10 μg per lane) probed with 1:1000 UT-Bc19 or 1:2000 NaKATP antibodies. **Key**: 1,4,7,10 = Anterior; 2,5,8,11 = Posterior; 3,6,9 = Cerebellum. [NOTE 1,2,3 = from rat 1; 4,5,6 = from rat 2; 7,8,9 = from rat 3; 10,11 = from rat 4.] **(C)** Bar graphs summarizing the densitometric analysis of the western blotting experiments comparing the three brain regions.

Western blotting experiments using UT-Bc19 antibodies were conducted to investigate UT-B protein expression in anterior, posterior and cerebellum brain tissue samples. Firstly, peptide inhibition experiments for the UT-Bc19 antibody were performed using samples from the three regions of the same rat brain (Fig. 2A). This was used to confirm specificity of any signals seen in the rat anterior, posterior, and cerebellum brain. The expected 30 & 35 kDa UT-B signals that were detected in experiments were less visible when the UT-Bc19 antibody was pre-incubated with 2 μg/ml specific immunizing peptide (SP), compared to a similar amount of a non-specific peptide (NSP). This was the case when both 1:1000 and 1:2000 hUTBc19 were used. However, a similar pattern was observed for any other signals detected, including a strong, distinct band at 100 kDa. These results confirmed all signals were caused by the antibodies raised against UT-B, rather than any antibody contamination. However, this did not determine that all signals detected represented UT-B proteins.

Analysis was repeated using samples from four different rats, with no pre-incubation of the UT-Bc19 antibody (Fig. 2B). A strong 35 kDa and weaker 30 kDa signal was observed in all samples, which did not differ in between brain regions. In contrast, the 100 kDa signal appeared relatively strongest in samples from anterior tissue, moderate in posterior tissue and weakest in cerebellum tissue. Alternatively, signals at 100 kDa corresponding to the expression of NaKATP, along with total protein loading, both appeared equal across all samples. As expected, densitometric analysis of signal-to-protein ratios confirmed no region-specific difference in abundance of either the 35 & 30 kDa UT-B (NS, N = 3–4, ANOVA) or the 100 kDa NaKATP (NS, N = 3–4, ANOVA) (Fig. 2C). In contrast, there was a very significant difference in the 100 kDa signal obtained with UT-Bc19 antibody (P < 0.001, N = 3–4, ANOVA). Post-hoc analyses using Tukey's HSD showed significant difference of abundance of non-UT-B 100 kDa normalized to NaKATP: P < 0.05 for Anterior vs Posterior; P < 0.01 Posterior vs Cerebellum; P < 0.001 for Anterior vs Cerebellum.

**Fig. 3:**
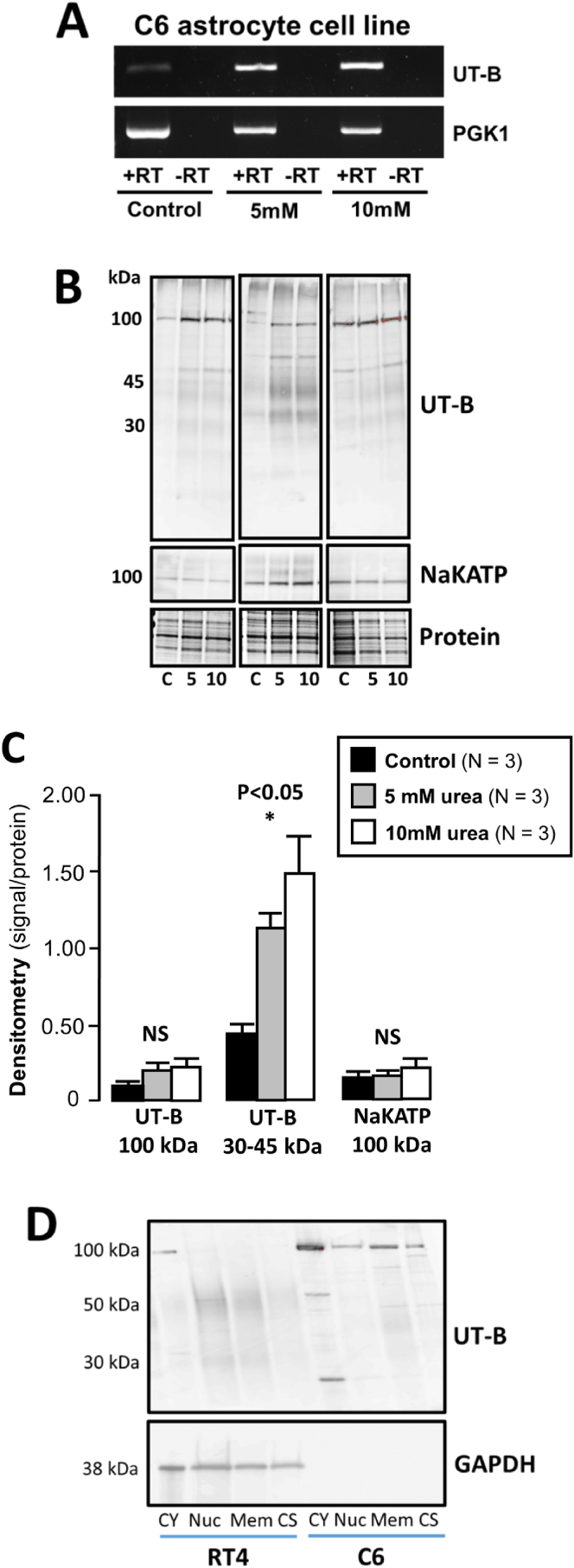
**Investigation of UT-B in the C6 rat astrocyte cell line. (A)** Endpoint RT-PCR experiments suggested that 48-h exposure to either 5 or 10 mM urea increased UT-B expression. **(B)** Western blots of membrane-enriched C6 samples showed that 48-h urea treatment increases 30 & 45 kDa UT-B signals. **(C)** Densitometric analysis showed the change in 30 & 45 kDa proteins was statistically significant, while no such changes occurred in the 100 kDa signals detected with either UT-Bc19 or NAKATP antibodies. **(D)** Western blots of compartmentalized protein samples from C6 and RT4 cells. UT-B detected 30–50 kDa signals in membrane-enriched samples, while the 100 kDa signal was predominantly in cytosolic samples. **Key:** CY = cytosolic; Nuc = Nuclear; Mem = Membrane; CS = cytoskeletal.

To further investigate the nature of the signals obtained in the rat brain tissue with the UT-Bc19 antibodies, additional experiments were performed using the C6 rat astrocyte cell line. Endpoint RT-PCR showed that UT-B RNA was weakly expressed in naive C6 cells but was upregulated by 48-h incubation in either 5 or 10 mM urea (Fig. 3A). Moreover, 48-h exposure to 5 or 10 mM urea was shown to increase the expression of 30 & 45 kDa proteins detected in membrane-enriched C6 cell lysates by UT-Bc19 (Fig. 3B) in a statistically significant manner (P < 0.05, N = 3, Repeated ANOVA) (Fig. 3C). In contrast, no significant changes were observed for the 100 kDa signals detected with either UT-Bc19 or the NaKATP antibodies (NS, N = 3, Repeated ANOVA). To further explore subcellular localization, protein expression analysis was carried out in membrane-, nuclear- and cytoplasmic-enriched C6 cell fractions (Fig. 3D). A weak, smeared signal was detected at 30–45 kDa in the membrane-enriched sample from un-treated C6 cells in response to the UT-Bc19 antibody, but no signal was observed in nuclear-enriched sample. In contrast, expression at 100 kDa was mainly detected in the cytoplasmic-enriched portion. RT4 bladder cells have previously been reported for their robust expression of UT-B [25]. Here, we reveal similar findings in the RT4 cells, expressing strong 30–50 kDa UT-B signals in both nuclear and membrane samples, while a weak signal at 100 kDa was observed solely in the cytosolic region of RT4 cells. GAPDH expression was used to indicate equal loading of RT4 cell lysates. Interestingly, GAPDH did not appear to be abundantly expressed within our C6 cell population (Fig. 3D).

**Fig. 4:**
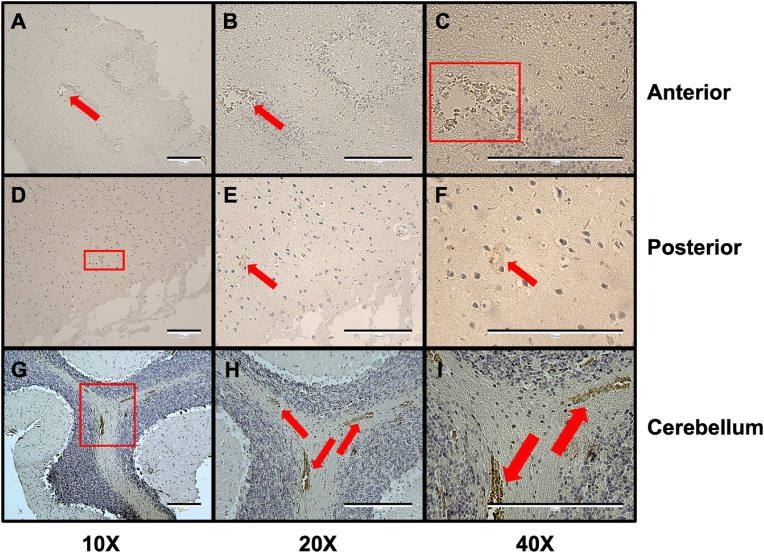
**Immunolocalization using UT-Bc19 on rat anterior, posterior, and cerebellum brain sections.** Rat anterior, posterior, and cerebellum brain sections (5 μm thickness) were respectively probed with 1:250 UT-Bc19, 1:100 UT-Bc19, and 1:250 UT-Bc19 antibodies. Images shown illustrate the potential UT-B staining in each region under magnification 10X, 20X, and 40X (scale bar = 200 μm), which is further highlighted using red box-shaped structures or arrows.

Having confirmed the expression of UT-B across rat brain regions, we sought to better identify its tissue-specific localization. Immunolocalization experiments were performed in 5 μm sections of rat anterior, posterior and cerebellum tissue using UT-Bc19 (Fig. 4). The staining revealed strong detection of UT-B in the plasma membrane of the red blood cells within blood vessels, which appeared particularly enriched in the cerebellum. Since UT-B plays a prominent role in red blood cells as a regulator of both urea and water transport [26], this was expected. In contrast, and somewhat surprisingly given the western blotting data, the detection of UT-B immunolocalization signals elsewhere was much less consistent, with no defined structures being regularly stained.

## 4 Discussion

Initially, RT-PCR experiments confirmed similar expression of UT-B RNA throughout in rat anterior, posterior, and cerebellum regions of the rat brain. Western blotting analysis using our well-characterized UT-Bc19 antibody [24] then confirmed the presence of 30 & 35 kDa UT-B signals, which were also equally distributed. These signals were very similar to those previously reported in the rat brain by other investigators, which identified both non-glycosylated 29 kDa and N-glycosylated 32.5 kDa UT-B1 proteins in rat brain using C-terminal polyclonal antibodies [22,23]. However, our UTBc19 antibodies also detected an accompanying 100 kDa signal. Based on our current findings we strongly believe this larger signal is not a UT-B protein. Firstly, the size of this band is much larger than would be predicted for UT-B. Secondly, we identified significant region-specific differences in expression, unlike the UT-B RNA expression or abundance for the 35 kDa protein. Thirdly, this 100 kDa signal was not upregulated in C6 cells by exposure to urea, as would be expected for a UT-B protein. Finally, the 100 kDa was predominantly found in cytosolic fractions for both C6 and RT4 cells, rather than the membrane fraction, and was weakly expressed in RT4 cells, which are known for their high level of UT-B protein [25]. These outcomes are supported by our previous studies that have reported similar 100 kDa bands in UT-B-deficient tissues, including the human liver [27]. Moreover, other groups’ investigations using similar antibodies targeted to the C-terminal of UT-B have observed similar 100 kDa signals in UT-B knockout mice [28,29]. Our interpretation of all these findings is therefore that the 100 kDa signal represents a non-UT-B protein that happens to have a similar shape to the C-terminal of UT-B. As a result, great caution must be taken whenever analyzing the results of detecting UT-B protein with antibodies raised against this epitope, including UTBc19.

This outcome is further consolidated by the lack of staining reported here in immunolocalization experiments, in which robust staining corresponding to UT-B was restricted the red blood cells within cerebellum blood vessels. Recent studies have detailed UT-B in rat brain in the third cerebral ventricular wall, granule cell layer of the dentate gyrus, and other parts of hippocampus, cerebral cortex, substantia nigra, habenular, and lateral hypothalamic nucleus [5]. Therefore, the lack of other specific staining patterns in the anterior, posterior and cerebellum regions in the current study was somewhat surprising. This difference suggests that our UTBc19 antibodies (i) may not detect rat UT-B protein as effectively as other species (e.g human), and (ii) are more effective for Western blotting rather than immunolocalization analysis. Indeed, this second observation has also been reported in a recent study on deer rumen samples using the UTBc19 antibodies [30]. Additionally, it should also be noted that all the animals investigated in this current study were male. As such, we cannot exclude the possibility that sex-differences might exist and are therefore worthy of future investigation.

Overall, this current study has shown that UT-B protein appears to be equally distributed across different regions of the rat brain. Moreover, our data shed light on the challenges of using antibodies targeted to the C-terminal of UT-B proteins. In particular, the difficulty of stating with confidence whether observed signals or staining is that of UT-B or a “non-UT-B″ protein – when clarity cannot necessarily be provided through specific peptide inhibition studies alone. It certainly highlights the caveats required when viewing reports interpreting immunolocalization findings with certainty on imagery alone, unless the use of UT-B knockout animals has been included [5]. Future studies investigating brain UT-B transporter involvement in human disease should therefore ensure that they investigate proteins with multiple techniques whenever possible – including large scale proteomic studies and western blotting, as well as immunolocalization.

## Declaration of competing interest

The authors declare that they have no known competing financial interests or personal relationships that could have appeared to influence the work reported in this paper.
